# Methodological modifications on quantification of phosphatidylethanol in blood from humans abusing alcohol, using high-performance liquid chromatography and evaporative light scattering detection

**DOI:** 10.1186/1471-2091-6-18

**Published:** 2005-09-27

**Authors:** Steina Aradottir, Bo L Olsson

**Affiliations:** 1 Department of Laboratory Medicine, Division of Clinical Chemistry and Pharmacology, Lund University, Lund University Hospital, S-221 85 Lund, Sweden; 2 AstraZeneca, R&D, Lund, Sweden

## Abstract

**Background:**

Phosphatidylethanol (PEth) is an abnormal phospholipid formed slowly in cell membranes by a transphosphatidylation reaction from phosphatidylcholine in the presence of ethanol and catalyzed by the enzyme phospholipase D. PEth in blood is a promising new marker of ethanol abuse depending on the high specificity and sensitivity of this marker. None of the biological markers used in clinical routine at the present time are sensitive and specific enough for the diagnosis of alcohol abuse.

The method for PEth analysis includes lipid extraction of whole blood, a one-hour HPLC separation of lipids and ELSD (evaporative light scattering) detection of PEth.

**Results:**

Methodological improvements are presented which comprise a simpler extraction procedure, the use of phosphatidylbutanol as internal standard and a new algorithm for evaluation of unknown samples. It is further demonstrated that equal test results are obtained with blood collected in standard test tubes with EDTA as with the previously used heparinized test tubes. The PEth content in blood samples is stable for three weeks in the refrigerator.

**Conclusion:**

Methodological changes make the method more suitable for routine laboratory use, lower the limit of quantification (LOQ) and improve precision.

## Background

The enzyme phospholipase D (PLD) normally catalyses the formation of phosphatidic acid from phosphatidylcholin using water as a substrate. In the presence of primary alcohols the reaction is diverted to transphosphatidylation and phosphatidyl alcohol is formed instead. As a consequence, human ethanol consumption leads to formation of phosphatidylethanol (PEth) in the tissues. In 1997 Hansson et al. published an article on measurements of PEth in whole blood extracts from alcoholics [1]. The separation method used was thin layer chromatography (TLC) and subsequently PEth was quantified by image analyses. It was demonstrated that PEth was measurable in blood from alcoholics several days, up to weeks, after the last alcohol intake. PEth was suggested to be a potential marker of alcohol abuse. The TLC separation followed by densitometric scanning was time consuming and had a relatively high coefficient of variation. To overcome these issues, a HPLC method optimised for separating PEth from other lipids in whole blood extracts was developed. PEth has physiochemical properties that differ considerably from other phospholipids, which make it possible to separate PEth as a single peak at safe distances from other peaks. An evaporative light scattering (ELSD) detector was used for detecting the lipids [2,3]. The ELSD detector quantifies any solute less volatile than the solvents. Functional groups, fatty acid chain-length or saturation has little or no effect on the detector response. However, a non-linear detector response following the quantitative principles of ELSD complicates the quantification. The method was optimized for measuring PEth in extracts from whole blood but the same performance parameters are also valid for quantification of PEth in extracts from organs [4-6].

In this paper, five methodological improvements of importance for usefulness and practicability are presented. First, the extraction procedure is simplified, second, it is shown that PEth can be analyzed in blood with EDTA as anticoagulant, third, the limit of quantification (LOQ) is lowered, fourth, an internal standard (IS) for the method is presented, and fifth, an improved method for calculation of PEth over a broad range is introduced.

## Results and discussion

### Extraction

Blood samples have in previous publications been extracted according to Radin [7] with 33 volumes of propan-2-ol:hexane, 3:2 v/v. In practice, this means that 300 μl blood can be extracted using 10 ml tubes. The use of 10 ml tubes is a limiting factor imposed by the standards in routine clinical laboratories. It was speculated that adding the blood first to propan-2-ol and thereafter adding the hexane, maintaining the same proportion of solvents, would make the extraction procedure more practical without negatively affecting the recovery of PEth. This variation of Radin's extraction method (Type II) was investigated and compared to the ordinary extraction according to Radin (Type I). Thus, in Type I extractions, blood was added to the premixed solvents, and in Type II extractions, blood was added to propan-2-ol during mixing before hexane was added. The same proportion (33 volumes) of solvent was used in both extraction methods. It was found that the stepwise addition of ingredients resulted in 25% higher recovery of PEth (p < 0.001, using 2-way analyses of variance (ANOVA), four patient samples were extracted in quintuple of each extraction type).

Beside of giving a higher recovery of Peth, the stepwise addition is easier to perform because less solvent is present in the tube when the blood is added. The reason for the higher recovery might be that cell membranes are disrupted and the lipid-protein linkages are broken by the propan-2-ol, which makes the phospholipids from the cell membrane more available than when hexane is present from the start. Recovery studies of extraction procedures for PEth have not been performed because of problems in incorporating defined amounts of PEth in the same way in the membranes as PEth occurs in vivo.

### Internal standard technique

**Figure 1:**
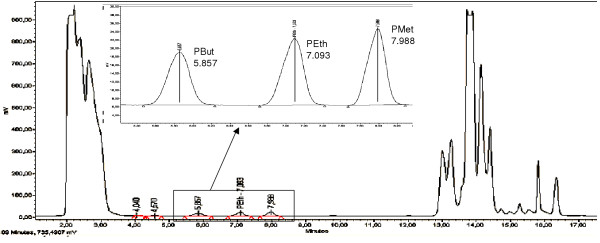
**HPLC chromatogram**. HPLC chromatogram of a 100 μl blood sample from abstainer. To the blood sample 2 nmol of each phosphatidylbutanol (PBut), phosphatidylethanol (PEth) and phosphatidylmethanol (PMet) was added. An expanded view is given in the insert.

Earlier attempts to find an internal standard (IS) for the PEth analysis method were not fruitful [8]. Internal standard methodology was judged to be an important improvement in quantification methodology because of its potential to correct for experimental variation (e.g., sample evaporation, injection volume and detector response). It was speculated that either phospatidylmethanol (PMet) or phosphatidylbutanol (PBut) might be suitable as internal standard because of their similarity to PEth and for not being endogenous lipids. The test run of PMet and PBut together with PEth standard (Fig 1) showed an Rf value for PBut at safe distances from both PEth and other peaks while PMet eluted at almost the same Rf value as carbamazepine, which is a drug that has anticonvulsant properties and is given to many alcoholic patients at hospitalisation. The dose response of PBut was very similar to that of PEth and by inspecting earlier runs of blood extracts from patients it was asserted that no endogenous compounds eluted at the same Rf value as PBut. It was decided to use PBut as internal standard for the PEth determinations. PBut did not interfere with endogenous lipids in blood.

**Figure 2:**
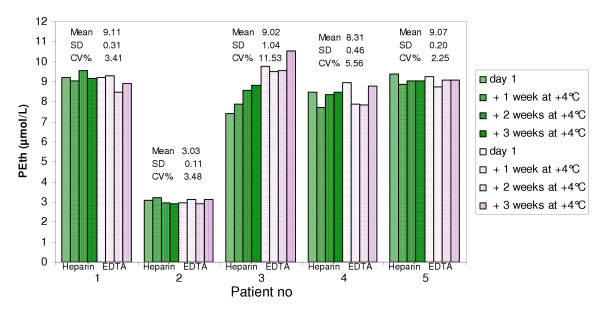
**Comparison of PEth content in heparin and EDTA blood during refrigerated storage**. Blood samples from 5 sober alcoholic patients was analysed for PEth amount on day 1 and after being stored in refrigerator for one, two and three weeks. No significant differences in PEth content could be found between heparin and EDTA blood at any time point (p = 0.33, 0.31, 0.77, 0.27, respectively, 2-way ANOVA). No significant changes in PEth content could be found during the three weeks of storage (p = 0.46, and 0.10 for heparin and EDTA respectively, 2-way ANOVA).

The extraction method was accommodated to internal standard use by adding 2.0 nmol of PBut to the propan-2-ol in the test-tube for unknown samples before blood and subsequently hexane were added. Because the detector response is non-linear, it is not optimal to use the simple IS evaluation technique where the same amount of IS is added to the unknown samples and the calibration samples, and where the calibration curve is constructed from the peak area ratios. A more accurate approach is to evaluate the apparent amount of both IS and analyte from separate standard curves and then apply the IS correction. Therefore, typical standard curves were prepared by adding both PEth and PBut in different amounts (e.g., 0.2, 0.5, 1.0, 2.0 and 4.0 nmol) to the propan-2-ol before blood and subsequently hexane were added. Aliquots of blood from abstainer were kept in a -20°C freezer and used for preparing standard curves; one aliquot was thawed for each preparation.

### Limit of quantification (LOQ)

The original extraction method [7] was developed to obtain good recovery of all lipids in blood. Since PEth is the only lipid of interest in the present method, it was investigated if smaller relative volumes for extraction would be adequate, because that would potentially improve the limit of quantification for PEth within the constraint of using 10 ml tubes. The extraction efficiency using 300, 600 and 900 μl blood and 10 ml extraction solution (corresponding to 33, 16.7 or 11 volumes of extraction solution) was investigated. Whole blood (300, 600 or 900 μl) was extracted by first adding blood to 4 ml of propan-2-ol containing IS, and then 6 ml of hexane, during agitation. After mixing twice, the samples were centrifuged at 1500 g for 10 min and the supernatants were transferred to new tubes. The PEth amount recovered per volume of blood was found to be independent of the relative volumes of extraction. The limit of quantification (LOQ) for the method has previously been determined to 0.2 nmol of PEth [9]. By increasing the amount of blood that can be extracted for a sample by 3 times, the LOQ, expressed in terms of whole blood concentration, was therefore lowered from 0.67 to 0.22 μmol/l, without making the extraction procedure more complicated. The modified extraction procedure was validated by extracting 26 patient samples using 300, 600 and 900 μl whole blood and 10 ml solvent. The result of a 2-way ANOVA of log-transformed values of PEth blood concentration showed no significant difference (p = 0.24) between the three treatment groups. The PEth blood concentration for these patients varied between 0.5 and 12 μmol/l.

### Comparison of PEth values in blood with different anticoagulants added

Blood from five alcoholic patients, being treated for alcohol abstinence, was drawn into both heparin and EDTA tubes. All patients had reached zero ethanol in expired air. The PEth amount in the samples was analyzed on day one and thereafter the samples were kept refrigerated and analyzed 1, 2 and 3 weeks after sampling. The PEth amount in the samples was not significantly different between heparin and EDTA blood at any time point (p = 0.33, 0.31, 0.77, 0.27 initially and after 1, 2, and 3 weeks of storage, respectively, using 2-way ANOVA). Furthermore, the PEth amount in the samples did not change during three weeks of refrigerated storage (Fig. 2, p = 0.46 and 0.10 for heparin and EDTA, respectively, 2-way ANOVA).

**Figure 3:**
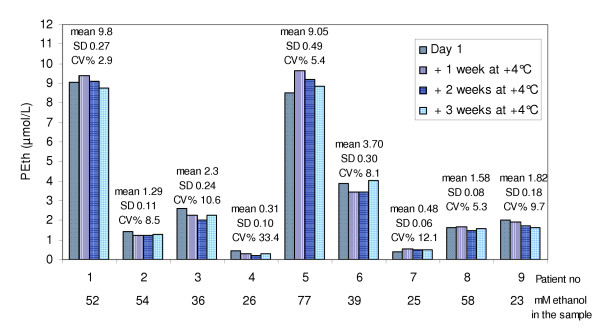
**Refrigerated storage of blood with ethanol present**. Blood samples from nine ethanol intoxicated patients analysed for PEth amount on day 1 and after being stored in refrigerator for one, two and three weeks. No significant changes in PEth content could be found during the three weeks of storage (p = 0.37, 2-way ANOVA).

The ability to use EDTA blood tubes for sampling makes the analyses more user-friendly since whole blood for haematological test is routinely collected in EDTA tubes that are not centrifuged, while most routine tests run on blood sampled in heparinized tubes use the plasma or serum after centrifugation, which causes a risk for wrong handling of the sample. In order to study possible PEth formation in vitro in refrigerated blood samples containing ethanol, blood from nine ethanol-intoxicated patients was drawn into EDTA tubes. Samples were also taken for blood ethanol analyses. The mean blood ethanol concentration was 43 mmol/L (range 23–77 mmol/L). The PEth amount in the samples was analyzed on day one and thereafter the samples were kept refrigerated and analyzed 1, 2 and 3 weeks after sampling. No significant effect of storage time on PEth amount was found (Fig. 3, p = 0.37, 2-way ANOVA).

**Figure 4:**
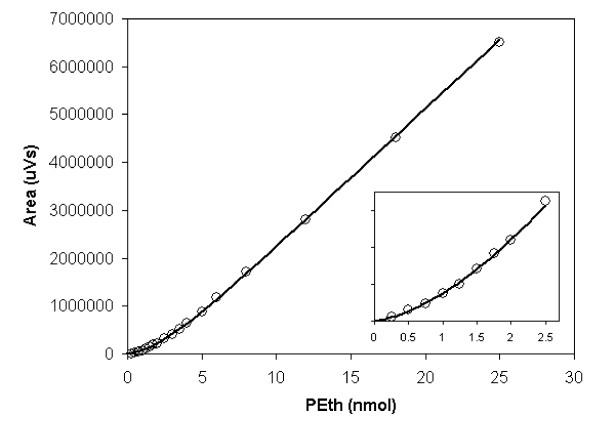
**Dose response curves**. Dose response curves of the ELSD detector to phosphatidylethanol. The solid line represents the fitted mixed linear-exponential function *y *= *a *+ *bx *- *a *exp(-*dx*). An expanded view of the low-amount area is given in the inset.

This stability of PEth in refrigerated samples with ethanol present has earlier only been investigated for a time period of three days [4]. Freezing blood at -20°C with ethanol present has been shown to highly elevate PEth levels whereas such samples can be frozen in liquid nitrogen and stored at -80°C without in vitro formation of PEth [4]. The latter method is, however, not practical in a routine setting. It is therefore important that the present investigation has demonstrated that samples can be stored refrigerated for an extended period of time with no in vitro formation of PEth, which in a routine laboratory is an advantage.

### Calibration and sample evaluation

According to the quantitative principles of ELSD, the detector response is non-linear. Curve fitting was therefore used to estimate the calibration function for evaluation of unknown samples. For the present application the detector response is exponentially shaped at lower amounts of PEth and gradually becomes linear at higher amounts. Exploiting this observation, a mixed linear-exponential function, *y *= *a *+ *bx *+ *c *exp(-*dx*), was tried and found to accurately trace the calibration data over the complete range of detector response. Here, y is the peak area and x the amount of PEth. The function can easily be reduced to a 3-parameter function by forcing it through the origin (by constraining *c *to equal -*a*). After substitution and rearrangement, the 3-parameter function becomes *y *= *a*(1-exp(-*dx*)) + *bx*. The reduction to a 3-parameter model that is bound to the origin is appropriate considering that the peak area is zero in the absence of Peth. In the present application further constraints are that the exponential component diminishes with growing *x*, that *y *increases monotonically with growing *x*, and that *x *and *y *are not negative. Therefore, the following numerical constraints apply in the present application: *a *≤ 0, *b *≥ 0, and *d *≤ -*b/a*. A standard Levenberg-Marquardt algorithm [10] was employed for the non-linear fitting procedure, which for numerical stability, was performed on scaled data. Initial estimates of *a *and *b *were obtained by ordinary linear least squares regression of *y *on *a *+ *bx*, while *d *was initially set to -*b/a *(largest ratio that maintain monotonicity). Then, in order to provide further robustness, the initial estimates were perturbed, one at a time, by a factor of two (up and down) and the best fit from the seven starting points was used as the final fit. An error model with weights taken as 1/*y *was used because variability is roughly proportional to the response.

To estimate the amount of PEth in an unknown sample (*x*) from the observed peak area (*y*), the inverse of the function was calculated numerically by an iterative range-halving method to a suitable precision defined as the absolute difference between observed *y *and *y *calculated from the function. This approach, of course, hinges on the constraint that the function is monotonously increasing with increasing *x*. Typically, the root-mean-square deviation between the fitted calibration function and the calibration data was about 3% over the range 0.25–25 nmol PEth (Fig. 4). The range of the curve fitting procedure used in earlier work [5,6] was limited to one order of magnitude.

### Evaluation of unknown samples with internal standard

With each batch of samples, a standard curve, evaluated using the mixed linear-exponential calibration function, was run. For every unknown sample the apparent amount of PBut was read from the standard curve for PBut and the inverse of this value was multiplied with the known amount of PBut added to the sample to give a sample correction factor. The apparent amount of PEth, obtained from the standard curve for PEth was then multiplied with this factor to obtain the corrected amount of PEth. The precision of the method was investigated by running duplicate samples at 4 different levels of PEth (3, 7, 10 and 15 μmol/l) together with a standard curve on 12 separate days. These samples had been frozen in aliquots of 250 μl and for each run, one aliquot was thawed and two extractions (100 μl each) were performed as described. Two different persons did the sample preparations, and the sample order was randomised during preparation and in the HPLC run. The within-day and between-day coefficients of variation (CV) were <6% and <12%, respectively.

**Figure 5:**
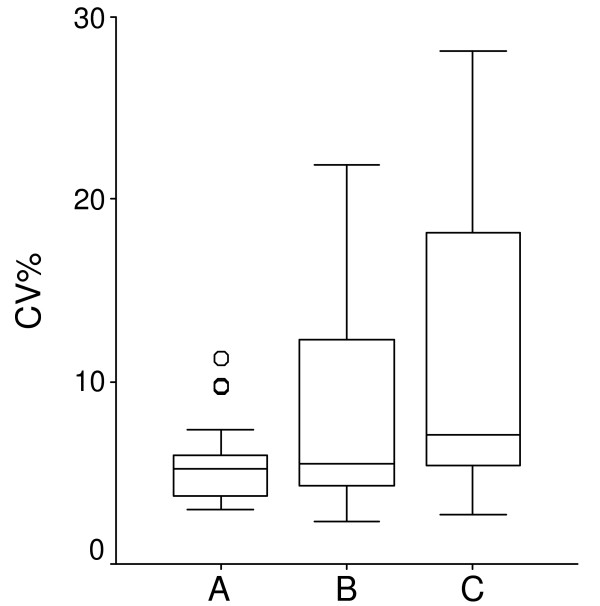
**The precision of the method**. Box plot of CV% of 26 patient samples. For each sample, extraction was done in duplicates of 300, 600, and 900 μl (sextet from each patient) analysed and evaluated in three different ways, **A **is evaluated with the new algorithm and internal standard (IS), **B **is evaluated with the new algorithm and **C **is evaluated with the old algorithm. The Box-plot identifies the median, the middle 50% of the data, the range, and the outliers (o).

The precision obtained using the IS methodology and the mixed linear-exponential calibration function (LinExp) (A) compared with LinExp without IS (B) and with the previous method (C) was validated as follows. Samples from 26 patients (with PEth concentration range between 0.6–11 μmol/L) were extracted in duplicates of each 300, 600, and 900 μl whole blood and were evaluated according to the three evaluation models (A, B, and C). Precision was calculated as the CV% for the 6 samples from each patient (Fig. 5). The precision obtained using the IS methodology and the LinExp (A) is several-fold better than obtained with LinExp without IS (B) or with the previous method (C). This clearly demonstrates the improved ruggedness provided by the IS methodology due to its potential to correct for experimental variation (e.g., sample evaporation, injection volume and detector response).

## Conclusion

Both heparinized and EDTA tubes are suitable as blood sampling tubes. Blood samples with and without ethanol present are stable in refrigerator for three weeks.

The extraction studies showed that more blood could be extracted with the same amount of extraction solution without sacrificing recovery, leading to a threefold improvement in the limit of quantification. The lowered LOQ makes it possible to detect intake of lower amount of ethanol than previously. The development of the internal standard methodology and the mixed linear-exponential calibration function considerably improved robustness, precision and measurable concentration range, all of which are important to facilitate routine application. The analytical method for PEth has been made more robust for use in the routine analytical laboratory.

## Methods

### Human sample collection

Peripheral blood samples were collected from patients abusing alcohol and healthy subjects into 4-ml standard blood sample tubes containing either sodium-heparin or EDTA as anticoagulant. The ethics committee of the Medical Faculty at Lund University, Sweden gave approval for this study (LU694-03).

### Chemicals

The solvents used for extraction and HPLC analysis (hexane, 1-propanol, propan-2-ol and triethylamine) were obtained from Merck (Darmstadt, Germany); all were of HPLC grade, except triethylamine which was of biochemistry grade. Acetic acid was obtained from BDH Laboratory Supplies (Pool, England) and was of HPLC grade. Deionized, sterile filtered water was obtained from a Millipore Milli-Q Plus water purification system and was checked regularly for conductivity. Ethanol was obtained from Kemetyl AB (Haninge, Sweden). Phosphatidylmethanol, phosphatidylethanol and phosphatidylbutanol were from Avanti Polar Lipids (Alabaster, USA). Sodium-heparin and EDTA tubes for blood withdrawal were of the brand Vacutainer^® ^(Becton Dickinson Vacutainer Systems Europe (UK)).

### HPLC analyses

The HPLC analysis was carried out with a Waters HPLC system Alliance 2695 with a thermostatted auto injector and an injection loop of 100 μl. A 250 × 4 mm, Licrosphere 100 DIOL, 5-μm particle size column (Merck, Germany) was used with a tertiary gradient of hexane (A), 1-propanol:water (17:3 v/v) (B) and 1-propanol:acetic acid:triethylamine (316:16:1 v/v) (C). A flow-rate of 1 ml/min and column temperature of 55°C was used, the gradient was run as described in Table 1. An evaporative light scattering detector Alltech 500 ELSD was used with nebulizer gas flow at 2.0 L/min and evaporator at 80°C. A computer-aided calculation of the peak area was performed with Waters MillenniumTM chromatographic software program.

**Table 1:** Gradient profile. Gradient profile used for the separation of PEth with the HPLC method.

	Mobile phase		
	
Time (min)	%A	%B	%C
0–3	85	1	14
3–8	85-63	1–23	14
8–12	63-36	23–50	14
12–13	36-0	50–86	14
13–23	0	86	14
23–28	0–85	86-1	14
28–58	85	1	14

A Hexane

B Propan-1-ol:water; 17:3 v/v

C Propan-1-ol:acetic acid:triethylamin; 316:16:1 v/v

Each sample has a total HPLC analysis time of 1 hour yielding a sample throughput of 24 per day using an auto injector. Attempts to increase the sample throughput, which would be of practical benefit in the routine setting has yet not been successful.

## List of abbreviations

ANOVA Analyses of Variance

EDTA Ethylene Diamine Tetraacetic Acid

ELSD Evaporative Light Scattering Detector

IS Internal Standard

LinExp Linear-Exponential calibration function

LOQ Limit of Quantification

PBut Phosphatidylbutanol

PEth Phosphatidylethanol

PMet Phosphatidylmethanol

TLC Thin Layer Chromatography

## Authors' contributions

SA contributed the subject area expertise, planned the study design, carried out all the experimental work and drafted the manuscript. BO contributed the conception of the calibration function and internal standard methodology and helped with the statistical evaluations and the drafting of the manuscript. This work is not associated with the professional affiliation of BO.
